# Do Web-based Mental Health Literacy Interventions Improve the Mental Health Literacy of Adult Consumers? Results From a Systematic Review

**DOI:** 10.2196/jmir.5463

**Published:** 2016-06-20

**Authors:** Bianca Brijnath, Joanne Protheroe, Kamal Ram Mahtani, Josefine Antoniades

**Affiliations:** ^1^ Curtin University School of Occupational Therapy and Social Work Perth Australia; ^2^ Institute of Primary Care and Health Sciences Arthritis Research UK Primary Care Centre Keele University Oxford United Kingdom; ^3^ Nuffield Department of Primary Care Health Sciences University of Oxford Oxford United Kingdom; ^4^ Department of General Practice Faculty of Medicine, Nursing and Health Sciences Monash University Notting Hill Australia

**Keywords:** health literacy, health care seeking behavior, Internet, intervention study, mental health, social stigma

## Abstract

**Background:**

Low levels of mental health literacy (MHL) have been identified as an important contributor to the mental health treatment gap. Interventions to improve MHL have used traditional media (eg, community talks, print media) and new platforms (eg, the Internet). Evaluations of interventions using conventional media show improvements in MHL improve community recognition of mental illness as well as knowledge, attitude, and intended behaviors toward people having mental illness. However, the potential of new media, such as the Internet, to enhance MHL has yet to be systematically evaluated.

**Objective:**

Study aims were twofold: (1) To systematically appraise the efficacy of Web-based interventions in improving MHL. (2) To establish if increases in MHL translated into improvement in individual health seeking and health outcomes as well as reductions in stigma toward people with mental illness.

**Methods:**

We conducted a systematic search and appraisal of all original research published between 2000 and 2015 that evaluated Web-based interventions to improve MHL. The PRISMA (Preferred Reporting Items for Systematic Reviews and Meta-Analyses) guidelines were used to report findings.

**Results:**

Fourteen studies were included: 10 randomized controlled trials and 4 quasi-experimental studies. Seven studies were conducted in Australia. A variety of Web-based interventions were identified ranging from linear, static websites to highly interactive interventions such as social media games. Some Web-based interventions were specifically designed for people living with mental illness whereas others were applicable to the general population. Interventions were more likely to be successful if they included “active ingredients” such as a structured program, were tailored to specific populations, delivered evidenced-based content, and promoted interactivity and experiential learning.

**Conclusions:**

Web-based interventions targeting MHL are more likely to be successful if they include active ingredients. Improvements in MHL see concomitant improvements in health outcomes, especially for individuals with mild to moderate depression. The most promising interventions suited to this cohort appear to be MoodGYM and BluePages, 2 interventions from Australia. However, the relationship between MHL and formal and informal help seeking is less clear; self-stigma appears to be an important mediator with results showing that despite improvements in MHL and community attitudes to mental illness, individuals with mental illness still seek help at relatively low rates. Overall, the Internet is a viable method to improve MHL. Future studies could explore how new technology interfaces (eg, mobile phones vs computers) can help improve MHL, mental health outcomes, and reduce stigma.

## Introduction

Despite the high global prevalence of mental illness [1], a significant treatment gap remains between those requiring care and those receiving care. In high-income English-speaking countries, such as the United States, the United Kingdom, and Australia, the prevalence of mental illness ranges from 14.9% to 24.6%, yet the treatment gap is 40%-65% [2-4]. One of the main reasons for this gap is low levels of mental health literacy (MHL) [5,6]. Defined as “the knowledge and beliefs about mental disorders, which aids their recognition, management or prevention” [7], MHL consists of 6 components: (1) the ability to recognize mental illnesses; (2) knowledge and beliefs about risk factors and causes; (3) knowledge about self-help interventions; (4) knowledge and beliefs about professional help available; (5) attitudes that facilitate recognition and appropriate help seeking; and (6) knowledge about how to seek appropriate mental health information [8].

Several studies have now conclusively shown that improvements in MHL improve community recognition of mental illness as well as knowledge, attitude, and intended behaviors toward people having mental illness [6,9-13]. The relationship between MHL and reduction in stigma toward people living with mental illness is still unclear [13]. However, these results have mainly been derived from evaluations of large-scale community mental health awareness campaigns delivered through traditional media such as television, radio, and print media; interpersonal contact with a person with a mental illness; and public seminars and community talks [6,9-13]. The potential of new media, such as the Internet, to enhance MHL has also been explored, but the actual effect on increasing MHL has yet to be systematically evaluated. Given the high rate of Internet penetration in the general population—more than 80% in the developed world—the Internet is an ideal medium through which to reach significant numbers of people at relatively low cost [14].

Currently, many MHL interventions are embedded within other interventions and it is difficult to disentangle the efficacy of each component of the intervention from the others (for review of interventions see [6,15]). However, identifying the efficacy of Web-based MHL interventions is important to enable a more strategic scale-up of what actually works and to increase the cost-effectiveness of any future intervention by discarding unsuccessful elements. A rigorous evidence-based cost-effective MHL intervention if successfully delivered via the web can harness the potential of the Internet, thereby increasing MHL at the population level and potentially delivering significant improvements in mental health outcomes among those living with mental illness.

To facilitate the development of such an intervention, the aims of this systematic review were twofold: (1) to collate the existing evidence to establish the efficacy of Web-based interventions that seek to improve MHL and (2) to establish, where possible, whether improvements in MHL translate into improvements in individual health seeking, reductions in stigmatizing attitudes toward people living with mental illness, and better health outcomes for individuals living with mental illness.

## Methods

### Search Strategy

This review was conducted in accordance with the PRISMA (Preferred Reporting Items for Systematic Reviews and Meta-Analyses) guidelines [16]. To identify eligible studies 6 databases were searched: PsycINFO, EMBASE, PubMed, CINAHL, and Web of Science. The search was conducted from April to August 2015 and search results were limited to English language, peer-reviewed articles published between 2000 and 2015. We did not search for articles published before 2000 because the global Internet penetration was only 6.5% at that time and we did not envisage any Web-based interventions targeting MHL before 2000. Key terms to identify studies included the following: Mental health literacy (Mental health, mental illness*, mental disorder*, mental disease*, depression AND literacy/*health literacy) and Internet (internet* or online* or web or World Wide Web or social media or website or surfing). Articles identified by the database search were screened to assess relevance to the aims. In addition, Google Scholar and selected reference lists were also searched to identify additional studies of interest. This review is a registered PROSPERO review: CRD42015025572.

### Study Inclusion and Exclusion Criteria

To be included, studies were required to meet the following criteria: (1) published in English in a peer-reviewed journal, (2) described a Web-based intervention with either the primary or secondary aim to improve MHL and included a measure of MHL or a component thereof such as mental health knowledge, (3) reported original research of a quantitative, qualitative, or mixed methods design, (4) included participants who were 17 years or older, and (5) included community members, family members and carers, and/or patients. Publications were excluded if participants in the study were health care professionals, the publications comprised commentaries or editorials rather than empirical research, or were published before 2000.

### Selection of Studies

Two authors (BB and JA) independently assessed relevant titles and abstracts. Selected studies were obtained in full text and reviewed in detail by 3 authors (BB, JA, and JP). Where any discrepancies arose, a consensus was reached through discussion or, if necessary, referral to another author (KM). After full-text review a number of studies were excluded as being irrelevant and the final number of included studies was obtained.

### Data Extraction

Data extraction was carried out independently by 3 authors (BB, JA, and JP). Where any discrepancies arose they were referred to a fourth author (KM). Data were extracted onto a predesigned worksheet relevant to our outcomes of interest.

### Quality Assessment

To ensure methodological rigor in the review process, all included studies were appraised by a minimum of 2 authors (JA, BB, or JP) for quality in accordance with the United Kingdom’s National Institute for Health and Care Excellence (NICE) guidelines and methodology [17]. As it is difficult to blind participants for behavioral treatment, we redefined the criterion regarding the blinding of participants. If blinding was not feasible, item 4 of the quality assessment was scored positive (+) if the credibility of the treatments was evaluated and treatments were equally credible and acceptable to participants; that is, control as well as intervention could be perceived to be an intervention in its own right [18].

## Results

**Figure 1:**
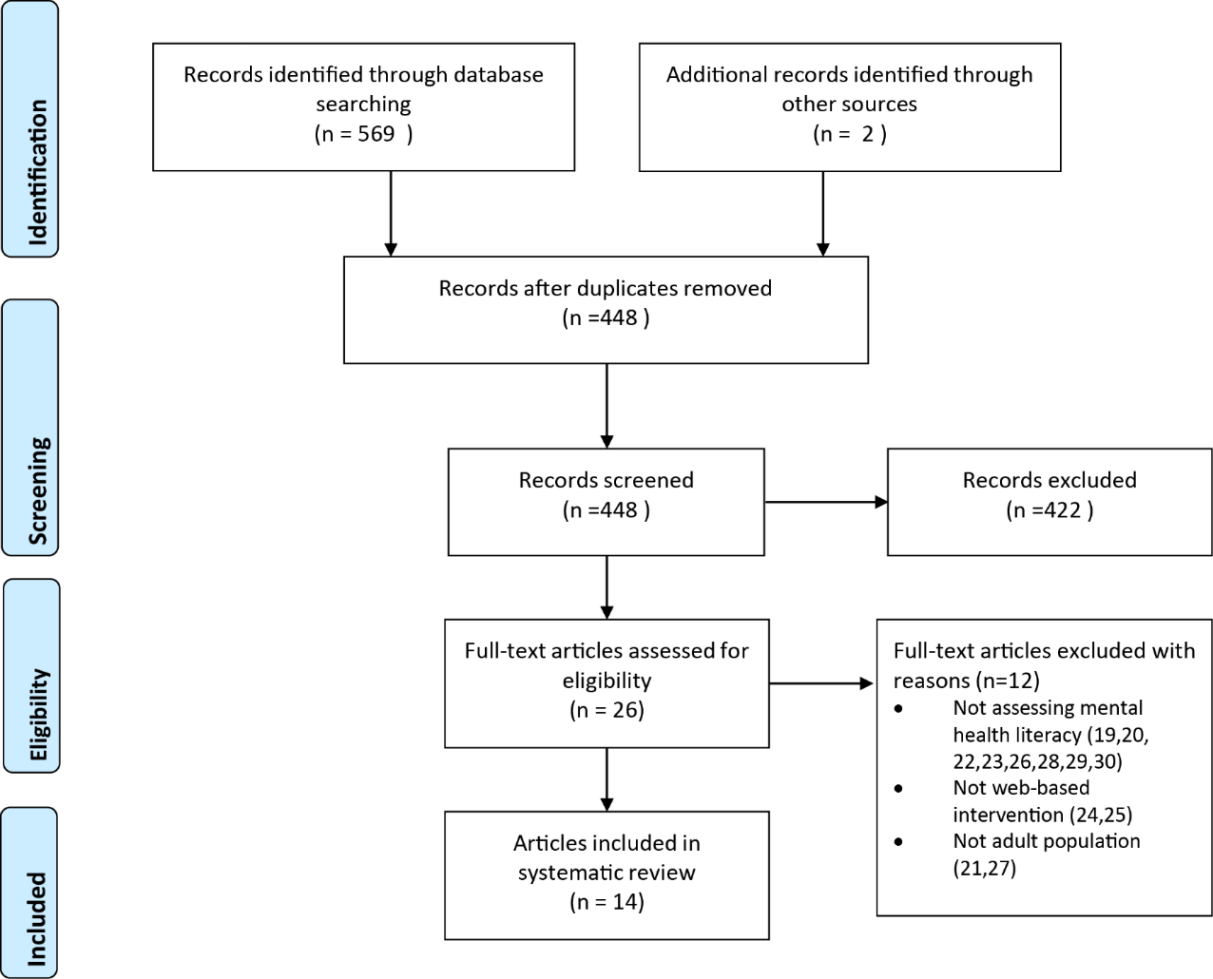
PRISMA flowchart.

Through the literature search, 571 potential records were identified (Figure 1); however, after the removal of duplicates, 448 studies were included for review based on title and abstract alone. Of the 448 studies, 26 were retained for full-text review. Full-text articles were reviewed by a minimum of 2 reviewers (BB, JA, JP) and were assessed for suitability for inclusion in accordance with the inclusion and exclusion criteria. During this process a further 12 papers were excluded as they did not meet the inclusion criteria of this review [19-30] (see Figure 1 – PRISMA flowchart for reasons). Therefore 14 articles were retained for inclusion [31-44]. Of these 14 papers, 2 papers reported on the same large randomized controlled trial (RCT) [31,36] but reported on different outcomes and were included as separate papers. However, this has been taken into consideration in the analysis for this review. The interrater agreement of the quality assessment was 84% and any disagreement between assessments after full-text review was resolved through consensus.

### Types of Studies

Of the 14 included studies, 10 were RCTs [31-34,36-38,40,41,44] and 4 were repeated-measures studies [35,39,42,43]. Two articles reported on the same trial [31,36], 7 studies were conducted in Australia [31,32,34,36-38,43,44], 4 studies were from the United States [33,35,41,42], 1 study was conducted in Hong Kong [39], and finally 1 study was Norwegian [40] but was reporting a trial of Australian self-help interventions, MoodGYM and BluePages translated into Norwegian. None of the reviewed studies included a qualitative exploration of the effect of the intervention on MHL, health seeking, stigma, or health outcomes. Five of the studies were complex interventions comprising 2 or more components [31,34,36,37,40].

### Participant Characteristics

Across the 14 studies the total pool of participants was 2605 individuals. Most studies included adult participants with clinical indication of a mental illness [32,33,35,37-39,42-44], and only 4 studies (5 papers) specifically recruited participants with mental health problems [31,34,36,40,41]. Two studies [33,42] specifically focused on family members and carers, 7 on the general community [32,35,37-39,43,44], and 1 study had a combined focus on patients and carers [41] (Multimedia Appendix 1). Despite the heterogeneity of target populations, comparability within and across groups was possible because most used the same constructs and measures; 7 studies used the Depression Literacy Questionnaire (D-Lit) alone or in combination with others to measure MHL [31,34-38,44]; 4 of the 6 studies reporting on stigma used the Depression Stigma Scale (DSS) [34,37,38,44] alone or in combination with other scales; 3 of the 5 studies reporting on help seeking used the General Help-Seeking Questionnaire (GHSQ) [32,37,44]; and 10 studies that included a measure of mental illness symptomatology used the Center for Epidemiologic Studies-Depression (CES-D) [31,32,34-36,38,40,41,43,44]. Further information is detailed in Multimedia Appendix 1: Study overview and characteristics. Eleven studies reported unequal gender representation with an average of 67.9% females [31,32,35-37,39-44].

### Study Quality Indicators

**Figure 2:**
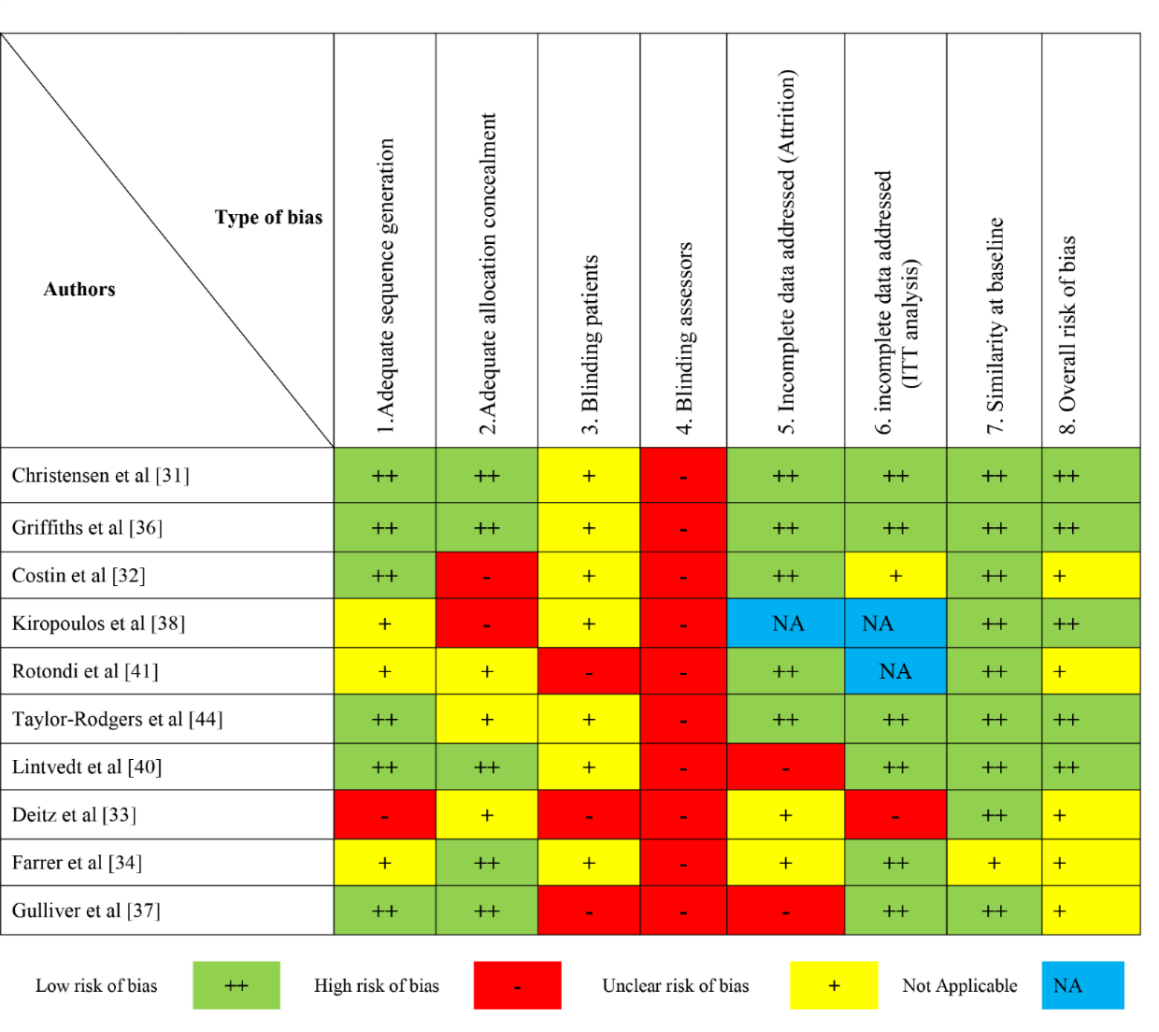
Risk of bias for randomized controlled trials.

A summary of risk of bias and quality indicators for RCTs can be found in Figure 2 and Table 1, respectively, and Table 2 for quality indicators and risk of bias in non-RCTs. Using the NICE guidelines to assess study quality, we found that a substantial number of studies reported high attrition rates (>20%) [31,33,34,37,39,40,43]; however, most studies included robust means of handling missing data such as intention-to-treat (ITT) analysis, which renders a conservative estimate of intervention effects [45]. Furthermore, although often practically unavoidable, some studies had small sample sizes, frequently related to recruitment and/or retention difficulties, and others recruited from limited pools of participants such as social clubs, student populations, or organizations, which may limit the generalizability of the findings (Tables 1 and 2). While blinding of participants is problematic for this type of intervention, which we have taken into consideration, most studies did not blind investigators who were involved with assessing the data, which could introduce detection bias. Moreover, in included RCTs randomization procedures were not consistently reported (Figure 2) and although the results of many of the included studies were encouraging, in some cases data were only collected immediately before and after the intervention with no subsequent follow-up, hence the sustainability of the interventions remains unclear (Table 1).

**Table 1:** Quality indicators and limitations for randomized controlled trials.

Authors	Recruitment	Data collection	Attrition	Adherence	Limitations
Christensen et al [31]	Election roll	Pre/post	Lost to follow-up: 18% for BluePages, 33% for MoodGYM, and 12% for control	Yes	Attrition rate Longer follow-up desirable
Griffiths et al [36]	Election roll	Pre/post	Lost to follow-up: 18% for BluePages, 33% for MoodGYM, and 12% for control	Not reported, but reported in Christiansen et al [31]	Small effect sizes Attrition rates Longer follow-up desirable
Costin et al [32]	Election roll	Pre/post (3 weeks after intervention)	Control (high/low distress): 14.5% Intervention (basic): 15.3% Intervention (enhanced): 17%	Yes	Power calculations suggest larger sample required No follow-up
Kiropoulos et al [38]	Welfare and social groups	Pre/post/1 week	0% (one-off access to website)	Not applicable	Sample may not be representative Researcher present during intervention Longer follow-up desirable
Rotondi et al [41]	Community mental health centers inpatient units	Pre/post/3, 6, 12 months	Patients: 3% Carers: 17%	Yes, high adherence	Small sample size Face-to-face workshop before intervention
Taylor-Rodgers and Batterham [44]	University	Pre/post	Control: 18% Intervention: 15%	Yes, 65.4% of intervention and 70.4% of control viewed all 3 Web pages	Small sample size University-based sample Longer follow-up desirable
Lintvedt et al [40]	University	Pre/post/2 months	Control: 28% Intervention: 46.9%	Not reported	Attrition rate University sample Longer follow-up desirable
Deitz et al [33]	Employees in 1 worksite	Pre/post	Not adequately reported: given response rate for intervention: 96%, control: 98%, but 22% of total sample did not view Web-based material	Not reported	Could not monitor “dosage” of intervention Limited sample Longer follow-up desirable
Farrer et al [34]	Mental health support hotline (Lifeline)	Pre/post/6-12 months	31% at postintervention 41% at 6-month follow-up	Not reported	Small sample size Attrition Adherence not reported
Gulliver et al [37]	Sports organizations	Pre/intervention week 1-2/post/3-6 months	49.2% at follow-up	Not reported	Small sample Study underpowered Attrition

**Table 2:** Quality indicators for nonrandomized controlled trials.

Authors	Recruitment	Randomization	Blinding^a^	Data collection	Attrition	Missing data handling	Adherence	Limitations
Shandley et al [43]	Not reported	N/A^b^	N/A	Pre/post/2 months	Post: 42.1% Follow-up: 62.4%	ITT^c^	Yes	Attrition; Limited adherence
Finkelstein and Lapshin [35]	University medical school	N/A	N/A	Pre-post (immediate)	Not applicable/data collected immediate pre/post	N/A	N/A	Follow-up; University sample
Li et al [39]	University	N/A	N/A	Pre/post	Post: 42.1%	ITT	Not reported in detail	Attrition; Small, university sample
Roy et al [42]	Military services	N/A	N/A	Pre/post/optional at 10 days	Post: 0% (only 1 event of using website) Optional follow-up: (74.4%)	N/A	Not reported	Lack of reporting on methods; Short, optional follow-up

^a^ Blinding of participants and/or personnel.

^b^ N/A: not applicable.

^c^ ITT: intention-to-treat.

### Impact on Mental Health Literacy

Across the reviewed studies there were an assortment of Web-based interventions (Multimedia Appendix 1) that targeted MHL as a primary outcome. Five of these studies employed samples with no prerequisite of symptomatology of mental illness [33,38,39,42,44].

In an innovative study, Li et al [39] tested a social network game, “ Ching Ching Story,” specifically designed to improve knowledge about mental health problems and the results were encouraging with significant improvements in MHL (Multimedia Appendix 2). Also targeting young adults, Taylor-Rodgers and Batterham [44] assessed the efficacy of a 3-week psychoeducational intervention based on vignettes about mental health problems on MHL as well as stigma, and help-seeking attitudes and intentions (Multimedia Appendix 1). Results suggested that the intervention was moderately effective (*d*=0.65) in improving anxiety literacy but not depression or suicide literacy and that there was a moderate change (*d*=0.58) in help-seeking attitudes, in particular toward seeking help from primary care providers (*d*=0.53; Multimedia Appendix 2).

Targeting carers of children between the ages of 5 and 21 years, Deitz et al [33] reported significant increases in overall knowledge of mental health problems using a 32-item questionnaire on the knowledge of childhood depression and anxiety (*P*=.008) and improved self-efficacy using a 9-item questionnaire on treatment seeking self-efficacy in handling mental health problems in children (*P*=.001; Multimedia Appendix 2). These changes resulted from a narrated and interactive Web-based mental health program. However, the program created no change in attitudes to help seeking or toward mental health problems (Multimedia Appendix 2). Roy et al [42] reported improved posttraumatic stress disorder (PTSD) knowledge, as measured using a 25-item PTSD knowledge questionnaire, at postintervention assessment after the use of an educational website for PTSD for the families of military service members specifically designed to increase PTSD knowledge and thereby support for returned military personnel. However, the duration of the intervention was unclear and significant attrition at follow-up was reported (74.4%; Multimedia Appendix 1 and Table 2, respectively). Similarly, the results of an RCT of 3 Web-based interventions (Multimedia Appendix 1) aimed at improving help seeking in young athletes indicated significant improvements in depression and anxiety literacy levels (Hedges’ *g*=0.90 and 0.90, respectively) compared with all other conditions [37] (Multimedia Appendix 2).

Addressing an extensive gap in the literature, Kiropoulos et al [38] evaluated an Internet-based, multilingual depression information resource targeted at Greek and Italian migrants. The results were encouraging with significant improvements in depression literacy and personal stigma (Multimedia Appendix 2); however, as in other studies, the sustainability of the intervention needs further exploration because participants were only followed up 1 week after the intervention (Table 1).

Although MHL was not the primary aim of the intervention, Shandley et al [43] evaluated a Web-based, CBT-based gaming intervention “Reach Out Central” aimed at supporting mental health in young adults, in particular targeting males (Multimedia Appendix 1). Outcomes suggested significant increases in help-seeking willingness (*η*^2^=.06), particularly for women, and slight improvements in MHL, but only for female participants (Multimedia Appendix 2).

In an RCT testing personalized eHealth cards (Multimedia Appendix 1) to improve help seeking and MHL, no significant results were reported on help seeking or MHL measures. A higher, but nonsignificant, number of positive beliefs about formal help sources and therapy for depression were recorded in the intervention arm (Multimedia Appendix 2). On the other hand, Finkelstein and Lapshin [35] found that their interactive, Web-based educational intervention for depression stigma was not only effective in improving depression stigma, but also significantly increased depression literacy (through the assessment of knowledge and resistance to treatment; Multimedia Appendices 1 and 2).

Three studies investigated the effect of Web-based depression interventions on MHL in populations with elevated depressive symptoms [31,34,40] (Multimedia Appendix 1). Christensen et al [31] conducted a large-scale RCT investigating the effect of BluePages, a depression literacy website, and MoodGYM, a Web-based CBT intervention. Participants in both interventions were followed up on a weekly basis by the research team, providing measurements on depression symptomology, dysfunctional thoughts, and CBT literacy. As hypothesized, both interventions were effective in improving depression literacy relative to the control group. The depression literacy intervention was most effective compared with the CBT intervention and control arm in improving depression literacy; similarly, the CBT intervention was most efficacious in improving CBT literacy (Multimedia Appendix 2).

Lintvedt et al [40] also assessed the effectiveness of BluePages and MoodGYM in Norwegian in improving MHL around depression and CBT in a sample of Norwegian university students. However, in this instance there was no follow-up of participants. Participants were assigned to either the intervention condition, which included access to both self-help websites, or a control condition (waitlist). Results further support the efficacy of MoodGYM and BluePages; the intervention significantly improved depression and CBT literacy and decreased depressive symptoms across all outcome measures, even without the weekly tracking previously reported by Christensen et al [31] (Multimedia Appendix 2). In an Australian study of individuals with psychological distress a comparable paradigm was employed [34]; participants were allocated to a combination of MoodGYM (6 weeks) and BluePages (1 week) without tracking (weekly 10-minute counselor phone call), tracking only, or control condition (Multimedia Appendix 1). Although CBT literacy significantly improved in Web-intervention conditions (*d*=0.71 and 0.80 without and with tracking, respectively), overall the intervention did not render a significant improvement in depression literacy and stigma. There did appear to be a short-term improvement in depression literacy and stigma in Web-based conditions, but this improvement was not sustained at 12-month follow-up (Multimedia Appendix 2). As suggested by the authors, these results suggest a dose-dependent effect of the psychoeducational intervention (BluePages) given the success in other trials in which the exposure to intervention content was of a more substantial duration [31,40].

### Impact on Seeking Help for Mental Illness

One study reported positive outcomes for help-seeking behaviors [42] after Web-based interventions [42]. While not reporting details relating to the method of data collection, 57% of carers who returned to a PTSD psychoeducational website 10 days after intervention reported having taken action in facilitating help for family member with suspected PTSD, including discussing symptoms and encouraging family member to seek help [42] (Multimedia Appendix 2).

Conversely, 2 studies found no improvement in formal or informal help seeking: Costin et al [32] found no indication that eHealth cards improved help-seeking intentions or actual help seeking among young people; neither did a Web-based mental health program for parents improve attitudes toward help seeking [33] (Multimedia Appendix 2). Similarly, although significant improvements in anxiety and depression literacy were reported, Gulliver et al [37] found no significant effect of their Web-based interventions on help-seeking attitudes, intentions, or behaviors relative to controls (Multimedia Appendix 2).

### Impact on Stigma

A multilingual Internet-based psychoeducational intervention was found to be effective in reducing personal but not perceived depression stigma [38] (Multimedia Appendix 2). Furthermore, reduction in depression stigma at postintervention and anxiety stigma at the 3-month follow-up was observed in the MHL and destigmatization condition of a brief, fully automated Internet-based help-seeking intervention [37] (Multimedia Appendix 2). Conversely, Taylor-Rodgers and Batterham [44] did not report significant changes in depression or suicide stigma, but a significant decrease in anxiety stigma (effect size=0.65) relative to the control group after a Web-based psychoeducational intervention (Multimedia Appendix 2). Likewise, Farrer et al [34] reported no overall significant improvement in depression stigma in response to MoodGYM/BluePages with or without participant follow-up; however, stigma appeared to be reduced after the intervention in both intervention conditions but only sustained in intervention without follow-up at 6 months. By the 12-month follow-up the effect was not sustained in either intervention (Multimedia Appendix 2). Shandley et al [43] reported minor gender differences in stigmatizing attitudes, with women holding less stigmatizing attitudes compared with men, which were sustained from pre- to postintervention. Moreover, the intervention did not significantly change attitudes toward people with mental illness (Multimedia Appendix 2).

### Impact on Mental Health Outcomes

Several studies reported improvements in mental health outcomes after Web-based interventions, in particular in studies that focused on patients with mental health problems. For example, Christensen et al [31] found both MoodGYM and BluePages to be effective in reducing depression symptomology in patients with clinical level of depression both in ITT analysis, which is more robust in accounting for missing data (*d*=0.4, 0.4, and 0.1 for MoodGYM, BluePages, and control, respectively), and in participants who completed all measures (n=414), as also reported by Griffiths et al [36]. Results were particularly encouraging in participants with CES-D scores of 16+ (n=369; completers: 0.6, 0.5, and 0.1 and completers with CES-D of 16+: 0.9, 0.75, and 0.25 for MoodGYM, BluePages, and control, respectively; Multimedia Appendix 2). Supporting these findings, Lintvedt et al [40] also found that MoodGYM and BluePages were effective in reducing depressive symptoms (*d*=0.57 on CES-D scores) and negative thoughts (*d*=0.50; Multimedia Appendix 2). Similarly, patients with schizophrenia experienced decrease in positive symptoms as a result of engaging with a targeted Web-based psychoeducation (*d*= −0.88) [41] (Multimedia Appendix 2).

Four studies reported no significant effect of their respective Web-based psychoeducational interventions on mental illness symptomology [32,38,44], nor was depressive symptomology significantly improved after a psychoeducational gaming intervention [43]. However, these studies were not specifically targeting individuals with mental health problems (Multimedia Appendix 2).

## Discussion

### Key Findings

The aims of this systematic review were twofold: first, to synthesize the existing evidence to establish the efficacy of Web-based interventions that seek to improve MHL. Second, to establish whether improvements in MHL translated into improvements in individual health seeking, reductions in stigmatizing attitudes toward people living with mental illness, and better health outcomes for individuals with mental illness. Outcomes from the review demonstrate that Web-based interventions targeting MHL are generally efficacious when they include the following “active” ingredients: the intervention comprises a structured program where participants are guided through a series of sequential steps, targets specific population or consumer groups, delivers evidence-based content (such as CBT and/or psychoeducation, depending on the target population), and is underpinned by a pedagogical approach that promotes interactivity and experiential learning. Examples of these types of interventions include MoodGYM and social network games [31,34,36,39,40]. Conversely, from our review we observed that interventions that do not fully utilize the interactive potential of the Internet, and deliver generalist information to consumers using an unstructured, didactic approach, and/or where participants can navigate and access the website in any way they chose, are less successful in improving rates of MHL [32,43,44].

Several studies found positive associations between increased MHL and reduced symptomatology, especially for mild to moderate depression [31,36,40,41]. This suggests that Web-based interventions may be best suited to target people with less severe mental illness (eg, depression and anxiety) and that are of a mild to moderate nature (eg, mild to moderate depression rather than clinical depression). However, this finding should be cautiously interpreted as the studies making these findings included therapeutic components (such as CBT) alongside psychoeducational ones, and separating the effects of each is not possible.

Nevertheless, to date the most extensively tested interventions suited to people with mild to moderate depressive symptoms appear to be MoodGYM and BluePages. Initially developed and tested in Australia, the intervention has also been translated into Norwegian and tested in Norway [40]. These interventions have been rigorously evaluated using the “gold standard” RCT designs and generally reported improvements across a variety of measures of MHL [31,34,36,40] and symptomatology [31,36,40]. However, these interventions also have a high attrition rate on account of the time commitment required from participants (up to 6 hours), and researchers must carefully consider the merits of this approach in relation to their target population and particular mental illness. Interventions seeking to increase MHL in community members are unlikely to be successful using this intensive approach; likewise for patients whose mental illness precludes them from concentrating for long periods of time. As shown by Rotondi et al [41], there are other Internet-based interventions that may also hold promise for mental illnesses such as schizophrenia, yet this line of research requires further substantiation.

The relationship between increased MHL and reductions in stigmatizing attitudes is more complex. On the one hand, the evidence demonstrates a positive association between the two—as MHL increases, stigma decreases. On the other hand, this evidence is based on participants’ self-report measures and it is difficult to establish how such attitudinal shifts inform everyday practices around inclusion and discrimination toward people with mental illness. Moreover, this review found no relationship between improvements in MHL and increased help seeking, suggesting that better knowledge about mental illness does not necessarily translate into people seeking the therapeutic care they might need. Avoiding the stigma of mental illness is one of the main reasons for not seeking appropriate and timely help [46,47]. Further research is needed to exemplify the potentially paradoxical relationship between MHL and help seeking.

### Limitations of the Included Studies

Our findings are tempered by 4 limitations in the current evidence base. First, there was high variability between the studies on the duration of the exposure-response relationship. Some studies incorporated a sustained engagement between the participants and the intervention into their design, and followed up over a prolonged period of time (eg, 12 months) to test the durability of the intervention (Tables 1 and 2). Other studies only had a one-off interaction between participants and the intervention and followed up participants for a very limited period (eg, 1 week; Tables 1 and 2). Second, monitoring participant adherence for complex interventions of this nature is challenging. Whether delivered via the web or through traditional platforms, there are many confounding factors—for example, social, cognitive, and structural—that could compromise the study results. Third, as several of the studies were complex interventions comprising multiple components it was unclear which components created the effects and whether these effects were intended or not. Finally, as acknowledged by many of the studies’ authors, certain standard measures and techniques, such as ITT analysis, were not applied to the studies because of their small sample size. Thus the extent of the generalizability of many of the studies is not entirely clear.

### Limitations of This Review

This review is also not without limitations. Only articles in English were included, thereby excluding research published in other languages. In addition, while the utmost care was taken to perform a thorough search, failure to include searches on specific mental illnesses (eg, schizophrenia) and literacy meant that we might have missed some studies, including evidence from the gray literature. Furthermore, because of the heterogeneity of the measures and outcomes of the included studies a meta-analysis could not be performed, limiting the overall rigor of the review. Lastly, as several of the studies were complex interventions comprising several components it proved difficult to disentangle which components influenced our target outcomes specifically.

### Conclusions

To the best of our knowledge this is the first review to examine the efficacy of Web-based MHL interventions and to establish the relationship between these interventions and their effect on help seeking, stigma, and health outcomes. As our review demonstrates, there are several “active” ingredients to a successful Web-based intervention and, if properly implemented, these interventions can improve MHL and symptomatology among those with mild to moderate mental illness. Of greatest promise are the MoodGYM and BluePages interventions [31,34,36,40,41] that have proven to be not only efficacious but also cost-effective and culturally portable from Australia to Norway [40]. Future research could extend the utility of these interventions by testing their applicability in other country settings different from Australia.

Interestingly, much of the intervention research on MHL comes from Australia. For more than a decade now there have been several Australian public health campaigns addressing mental illness, nearly all of which have integrated components of MHL [10,48-52]. Australia is often cited as an exemplar in this field [13]. Much could also be learned from applying the lessons from Australian interventions in low- and middle-income countries, where Internet penetration is rapidly increasing [14]. Documenting how interventions are adopted and adapted to culturally diverse settings could open new horizons for scholarship vis-à-vis the relationship between MHL, help seeking, stigma, and health outcomes in culturally diverse settings. Finally, future studies could also explore how these relationships are influenced by the technology interface being used (eg, mobile phones vs computers). By realizing these future avenues for research, we can better harness the full potential of the Internet and new technologies in delivering new innovations to help improve the lives of people with mental illness.

## Appendix

Multimedia Appendix 1 Study overview and characteristics.

Multimedia Appendix 2 Study outcomes.

## Abbreviations

CBT: cognitive behavioral therapy
CES-D: Center for Epidemiologic Studies-Depression
ITT: intention-to-treat
MHL: mental health literacy
NICE: National Institute for Health and Care Excellence
PRISMA: Preferred Reporting Items for Systematic Reviews and Meta-Analyses
PTSD: posttraumatic stress disorder
RCT: randomized controlled trial
