# Supplementary material for: Do Web-based Mental Health Literacy Interventions Improve the Mental Health Literacy of Adult Consumers? Results From a Systematic Review
Source: J Med Internet Res. 2016 Jun 20;18(6):e165. doi: 10.2196/jmir.5463 (PMC4932246; doi:10.2196/jmir.5463)
Supplement: Multimedia Appendix 1 [file jmir_v18i6e165_app1.pdf]

Multimedia appendix 1 Study overview & characteristics

Authors	Design	Group	Intervention/duration	Static(S) Dynamic (D) Unclear (U)	Control/duration	Target	Total N	Outcome measures of interest
Christensen et al [31]	RCT	**Clinical sample:** Adults with depression (K10>22) (18-52 YO)	Blue pages (Psychoeducation) or MoodGYM (CBT) with weekly follow up by lay interviewer/ 5 weekly modules and 1 week of revision	BluePages:S MoodGYM: D	Placebo control condition/6 weeks	Depression & mental health knowledge	525	**Symptoms**:CES-D/ATQ **MHL:** (Depression, Medical, Psychological & Lifestyle literacy scales) D-Lit/CBT-Lit
Costin et al [32]	RCT	**Community sample:** Young adults (19-24 YO)	Health E-cards (depression information)/Enhanced Health E-cards(depression & help seeking information)/received email each week for 3 weeks	S	Placebo control condition/3 weeks	Help seeking for Depression	348	**Symptoms:** CES-D **MHL**: Beliefs and Knowledge about help seeking, recognition of depression (Based on Jorm 1997 [7] **Help seeking**: AHSQ , GHSQ
Deitz et al [33]	RCT	**Carers sample:** Working carers of children between 5-21 YO	Web-based youth mental health program for carers/2 weeks	D	wait list condition	Depression and anxiety knowledge and self-efficacy in parents	99	**MHL:** Knowledge of childhood depression and anxiety **Mental health attitudes:** ATTSPH & Attitudes about emotional and mental health problems in youth/ Treatment self-efficacy and confidence in ability to address mental health issues in children NOTE: additional scales employed not reported here

Authors	Design	Group	Intervention/duration	Static(S)<br>Dynamic (D)<br>Unclear (U)	Control/duration	Target	Total N	Outcome measures of interest
Farrer et al [34]	RCT	**Clinical sample:**<br>Individuals with depressive symptoms (K10>22) (age not reported)	**Web Only**<br>1 week psycho education (BluePages)/MoodGYM weeks 2-6<br>**Web with tracking**<br>Additional 10 min weekly counselling<br>**Tracking Only**<br>received 10 min counselling call weekly	BluePages:S<br>MoodGYM: D	wait list condition	Depression health outcomes including MHL measures	155	**Symptoms: K10**<br>**MHL:**D-LIT /CBT-LIT<br>**Stigma:** DSS<br>**Other:** Audit/ EUROHIS-QOL
Finkelstein, Lapshin [35]	Repeated measures	**Community sample:**<br>Staff and Students from medical school (*m*Age=31.8)	Depression stigma reduction (CO-ED web based system)<br>Duration not reported, appears to be once off	D	NA	Depression stigma	42	**Symptoms:** PHQ-9<br>**MHL:** DKS, D-LIT, RTS<br>**Stigma:** BSDS (MDD & HIV)
Griffiths et al [36]	RCT	**Clinical sample**:<br>Adults with depression (K10>22) (18-52 YO)	Blue pages (Psychoeducation) or MoodGYM (CBT) with weekly follow up	BluePages: S<br>MoodGYM: D	Attention control condition	Stigmatizing attitudes to depression	As [31] (sample not included in total N for review)	**Symptoms**: CES-D<br>**Stigma scale**: 18-item self constructed<br>**MHL**: D-lit/CBT-Lit
Gulliver et al [37]	RCT	**Community sample:**<br>Athletes<br>(18-48 YO)	**Intervention 1(I1)**<br>Web-based mental health literacy and destigmatization<br>**Intervention 2(I2)**<br>Depression and Anxiety Symptom feedback condition/I3: Minimal content (just list of help seeking resources)/2weeks	I1:S<br>I2:D	Placebo with emails to online measurement surveys	mental health help seeking behaviours and attitudes	59	**MHL:** D-Lit , A-Lit<br>**Help Seeking:** ATSPPH-SF GHSQ, AHSQ<br>**Stigma**: DSS, GASS
Kiropoulos et al [38]	RCT	**Community sample:**<br>Italian and Greek immigrants (48-88 YO)	Multilingual Depression information website (MID-online)/1.5 hr	S	depression interview	Depression literacy and stigma	202	**Symptoms:** BDI-II<br>**MHL** : D-Lit<br>**Stigma**: DSS

Authors	Design	Group	Intervention/duration	Static(S) Dynamic (D) Unclear (U)	Control/duration	Target	Total N	Outcome measures of interest
Li et al [39]	Repeated measures	**Community sample:** Healthy young adults (17-25 YO)	web-based social network game (facebook)/3 weeks	D	na	Mental health literacy (knowledge and problem solving)	127	**Mental health literacy** (knowledge and understanding of MH concepts and application of skills) not validated
Lintvedt et al [40]	RCT	**Clinical sample:** University students (K10>20) (Mage=28.2)	Internet-based self-help (Norwegian MoodGYM and BluePages available) /8 weeks	BluePages:S MoodGYM: D	wait list condition	Depression prevention	163	**Symptoms:** K10/CES-D / ATQ* **MHL:** Treatment Depression Literacy (TDL) (Depression, Medical, Psychological & Lifestyle literacy scales)/CBT-Lit
Rotondi et al [41]	RCT	**Clinical & carers sample:** Adult Schizophrenic patients & carers	Web-based psychoeducational intervention (4 hour pre-workshop on survival skills)/12 months	D (forums & expert advice)	Usual care	Schizophrenia symptomology and knowledge	55	**Symptoms:** SAPS **MHL**: KASI
Roy et al [42]	Repeated measures	**Family sample:** Adult military service members family	PTSD information online/reporting unclear appear to be a once-off session	U	na	PTSD literacy in family members (and help seeking actions but only mentioned)	497	**MHL**: PTSD knowledge questionnaire (Not described in detail **Help seeking:** measure not disclosed

Authors	Design	Group	Intervention/duration	Static(S) Dynamic (D) Unclear (U)	Control/duration	Target	Total N	Outcome measures of interest
Shandley et al [43]	Repeated measures	**Community sample:** Young adults (18-25 YO)	Online gaming program (Reach Out Central) to support mental health of young people/4 weeks	D	na	Mental health literacy, mental health stigma, and willingness to seek help	266	**Symptoms: K10** **MHL:** 1 question **Help seeking:** 10 point scale **Stigma:** Brief questionnaire (not described) **Other:** **Coping:** CSI-SF Re**silience:** RS **Alcohol use:** AUDIT **Life satisfaction:** SWLS
Taylor-Rodgers, Batterham [44]	RCT	**Community sample:** Young adults (18-25 YO)	Brief online psychoeducation/3 weeks	U (included vignettes unsure of presentation)	attention matched control condition (websites for general health and dental health)	Mental health literacy, stigma and help seeking attitudes and intentions for depression anxiety and suicide	67	**Symptoms**: GAD-7/PHQ-9 **MHL**: A-LIT/D-LIT/ Literacy of Suicide **Help seeking:** ATTSPH-SF/GHSQ **Stigma:** DSS/ GASS/SOSS

Symptomatology
CES-D Center for Epidemiologic Studies Depression Scale
ATQ Automatic Thoughts Questionnaire
BDI-II Beck Depression Inventory
SAPS Scale for Assessment of Positive Symptoms
PHQ-9 Patient Health Questionnaire 9
GAD-7 Generalized Anxiety Disorder 7
K10 Kessler Psychological Distress Scale

Mental health literacy
D-Lit Depression Literacy Questionnaire
CBT-Lit CBT Literacy questionnaire
KASI Knowledge About Schizophrenia Interview
A-Lit Anxiety Literacy Questionnaire
DKS Depression Knowledge Survey
RTS Resistance to Treatment Survey

Help seeking scales
AHSQ Actual Help Seeking Questionnaire
GHSQ General Help Seeking Questionnaire
ATTSPH-SF Attitudes Toward Seeking Professional Help-Short form

Stigma
DSS Depression Stigma Scale
GASS Generalized Anxiety Stigma Scale
SOSS Stigma of Suicide Scale
BSDS Bogardus Social Distance Scale

Other
CSI-SF Coping Strategies Inventory Short-Form
RS Resilience Scale
AUDIT Alcohol Use Disorders Identification Test
SWLS Satisfaction With Life Scale
EUROHIS-QOL European Health Interview Survey-Quality of Life
BSDS (MDD & HIV) Bogardus Social Distance scale (Major depression & HIV)
