# Supplementary material for: Do Web-based Mental Health Literacy Interventions Improve the Mental Health Literacy of Adult Consumers? Results From a Systematic Review
Source: J Med Internet Res. 2016 Jun 20;18(6):e165. doi: 10.2196/jmir.5463 (PMC4932246; doi:10.2196/jmir.5463)
Supplement: Multimedia Appendix 2 [file jmir_v18i6e165_app2.pdf]

Multimedia appendix 2 Study outcomes

Authors	Intervention	Outcome measures of interest	Mental Health Literacy sig.	Symptomatology sig.	Help seeking behaviours/intentions/attitudes sig?	Stigma sig
Christensen et al [31]	Blue pages or MoodGYM with weekly follow up by lay interviewer	**Symptoms**:CES-D/ATQ<br>**MHL:** (Depression, Medical, Psychological & Lifestyle literacy scales) D-Lit/CBT-Lit	**D-Lit: BluePages Vs Control & MoodGYM:** Yes, Significant improvement (P<.05)<br>**CBT Literacy:** MoodGYM VS BluePages & Control: Sig. Improvement (P<.05)	**ITT analysis:**<br>**CES-D**: BluePages Vs Control: Significant (CI, 95%=2.9* (0.6-5.2))<br>MoodGYMVs Cont: Significant (CI, 95%: 3.2* (.9-5.4))<br>**ATQ**:Significant MoodGYM Vs Con (CI, 95%: 6.1*(1.9-10.4)<br>**Effect sizes pre-post:**<br>MoodGYM : 0.4<br>BluePages : 0.4<br>Control : 0.1	NA	NA
Costin et al [32]	Health E-cards	**Symptoms:** CES-D<br>**MHL**:Beliefs and Knowledge about help seeking, recognition of depression (Based on Jorm 1997 (Insert ref))<br>**Help seeking**: AHSQ , GHSQ	**Mental Health Literacy:** No , except improved beliefs relating to formal help seeking (P=.02) as well as benefit of interpersonal psychotherapy (P<.001) across interventions	CES-D: Not significant	**AHSQ**: No<br>**(GHSQ)**: Partly (P<.001) (formal sources) in intervention groups	NA
Deitz et al [33]	Web-based youth mental health program for carers	**MHL:** Knowledge of childhood depression and anxiety<br>**Mental health attitudes:** ATTSPH & Attitudes about emotional and mental health problems in youth/ Treatment self efficacy and confidence in ability to address mental health issues in children<br>NOTE: additional scales employed not reported here	Yes, Increase in all Mental Health knowledge domains except parenting skills relative to control (f=7.43, p<.008) Significant differences between I and C in self efficacy in handling Mental Health problems (f=12.73, p=.000). No other significant results	na	No significant difference I Vs C on ATTSPH	na

Farrer et al [34]	**BluePages/MoodGYM with and without tracking**	**MHL:**D-LIT /CBT-LIT<br>**Stigma:** DSS<br>**Other:** Audit/ EUROHIS-QOL	D-Lit: Overall non significant omnibus, but some moderate to large effects sizes reported: Web Only (d=.31) and Web with tracking (d=.01) compared to control. Tracking only Vs Web Only (d=0.37) and Web with tracking (d=0.73)<br><br>CBT-LIT:Both web interventions significantly improved CBT Literacy. Web Only (d=.71) and Web with tracking (d=.80) compared to control. Tracking only Vs Web Only (d=0.92) and Web with tracking (d=1.03)	na	na	DSS: Non-significant interaction effects, but significant between condition outcomes: Web only (d=.94) and web with tracking (d=.17) compared to control, Web only (d=.96) and web with tracking (d=.24) compared to tracking only
Finkelstein, Lapshin [35]	Depression stigma reduction (CO-ED web based system)	**Symptoms:** PHQ-9<br>**MHL:** DKS, D-LIT, RTS<br>**Stigma:** BSDS (MDD & HIV)	Sig. increase in depression knowledge (p=.00008)	NA	NA	Internet-based education significantly decreased the level of depression stigma on all measures (BSDS-MDD 10.6 ± 4.4 versus 7.2 ± 4.4, p < 0.001; DSS-personal 12.7 ± 7.2 versus 7.8 ± 5.3, p < 0.001; DSS-perceived 21.7 ± 5.5 versus 12.4 ± 5.5, p < 0.001).
Griffiths et al [36]	Blue pages or MoodGYM with weekly follow up	**Symptoms**: CES-D<br>**Stigma scale**: 18-item self constructed<br>**MHL**: D-lit/CBT-Lit	Increase in D-lit scores in BluePages: D-lit=-4.28, p<0.001 MoodGYM:= -0.7, p<0.02 CBT lit Blue pages: -1.47, p<.001 and MoodGYM: -2.84, P<.001	Decrease in CES-D scores: 3.32, p<.001	NA	Small decrease in personal stigma, but not perceived stigma<br><br>**Effect sizes personal stigma (ITT)**; 0.12 (BluePages), 0.11 (MoodGYM), -0.07 (Control)<br>**Effect sizes perceived stigma**:

Gulliver et al [37]	Web-based mental health literacy and destigmatization/ Depression and Anxiety Symptom feedback condition	**MHL:** D-Lit , A-Lit<br>**Help Seeking:** ATSPPH-SF , GHSQ, AHSQ<br>**Stigma**:DSS, GASS	**D-LIT**: Significant interaction between condition and measurement occasion ($F_{6,69.41}$ = 2.47, P = .03). Effect sizes relative to control: mental health literacy/destigmatization condition (g = 0.90, 95% CI 0.05–1.75), feedback condition (g = 0.13, 95% CI –0.66 to 0.92), and help-seeking list condition (g = –0.34, 95% CI –1.11 to 0.44).<br>**A-LIT**: Sig. interaction between condition and measurement occasion ($F_{6,67.51}$ = 3.99, P = .002). Effect sizes relative to control: mental health literacy/destigmatization condition (g = 0.90, 95% CI 0.05–1.75), feedback condition (g = –0.33, 95% CI –1.12 to 0.47), and help-seeking list condition (g = –0.10, 95% CI –0.87 to 0.67).	NA (K10 used at intake, but not reported on in terms of outcomes)	Positive trend towards seeking help from formal sources in Mental Health literacy/destigmatization condition Vs. Control (p=.06)	**DSS:** mental health literacy/destigmatization condition decrease on D-Lit relative to all conditions from pre- to post.Between group effect size: mental health literacy/destigmatization condition (g = 0.25, 95% CI –0.57 to 1.06), feedback condition (g = –0.15, 95% CI –0.94 to 0.65), and help-seeking list condition (g = 0.26, 95% CI –0.51 to 1.04), but improvement was not sustained at 3 month follow-up.<br>**ASS**: Significant mental health literacy/destigmatization condition significant decrease in ASS from pre-to postintervention relative to the feedback condition only. At 3 months follow-up mental health literacy/destigmatization condition was superior to all conditions; mental health literacy/destigmatization condition (g = 0.50, 95% CI –0.41 to 1.41), feedback condition (g = 0.12, 95% CI –1.02 to 0.78), and help-seeking list condition (g = 0.04, 95% CI –0.78 to 0.87).

Kiropoulos et al [38]	Multilingual Depression information website (MID-online)	**Symproms: BDI-II**<br>**MHL** : D-Lit<br>**Stigma**: DSS	**D-Lit**: Yes, intervention Vs control: (P<.001), Significant pre-post improvement *d*=-1.78	BDI-II: Not significant	NA	DSS: Intervention Vs Control:Significant personal stigma (P<.001) Only: Pre-post: Personal stigma, Intervention:*d*=.83/Control:*d=.06*<br><br>Perceived stigma:Intervention: *d*=-.14/Control:*d*=.16
Li et al [39]	Web-based social network game (facebook)	**Mental health literacy** (knowledge and understanding of MH concepts and application of skills) not validated	Significant improvement (participant analysis (D=.65) and ITT (D=.66))	NA	NA	NA
Lintvedt et al [40]	internet-based self help (Norwegian MoodGYM and BluePages available)	**Symptoms:** K10/Ces-D / ATQ*<br>**MHL:** T**reatment Depression Literacy** (TDL)(Depression, Medical, Psychological & Lifestyle literacy scales)/CBT-Lit	Yes, significant between group differences in depression literacy (d=.56) (ITT analysis)	Yes, Ces-D: Improvement in intervention group (d=.57)/ATQ between groups sig. (d=.50)	na	na
Rotondi et al [41]	Web-based psychoeducational intervention (4 hour pre-workshop on survival skills)	**Symptoms:** SAPS<br>**MHL**: KASI	**Patients:** knowledge about diagnosis (d=.88) / not sig. other KASI domains relative to controls.<br>**Carers:** Sig. Improvement knowledge about prognosis (d=1.94) no other sig. effects	**Patients:** Improvement in positive symptoms (*d*= -.88)	NA	NA
Roy et al [42]	PTSD information online	**MHL**: PTSD knowledge questionnaire (Not described in detail	Significant improvement in PTSD knowledge (d=1.2). Interaction effect of education suggestion those with less education might benefit more	NA	Follow up suggest 57% had taken action to help service member	NA

Shandley et al [43]	Online gaming program (Reach Out Central)	**Symptoms: K10**<br>**MHL**: 1 question<br>**Help seeking:** 10 point scale<br>**Stigma:** Brief questionnaire (not described)<br>**Other:**<br>**Coping:** CSI-SF Re**silience:** RS<br>**Alcohol use:** AUDIT<br>**Life satisfaction:** SWLS	Slight increase in MHL for females reported (pre-intervention:87%/Post intervention: 90%)	not significant	Sig. increase in help seeking willingness (n2=0.06) particular for females	No significant difference
Taylor-Rodgers, Batterham [44]	Brief online psychoeducation	**Symptoms**: GAD-7/PHQ-9<br>**MHL**: A-LIT/D-LIT/ Literacy of Suicide<br>**Help seeking:** ATTSPH-SF/GHSQ<br>**Stigma:** DSS/ GASS/SOSS	**A-LIT:** Sig. between group effect (d=.65).<br>**D-LIT or Literacy of suicide**: Not Sig.	**GAD-7 or PHQ-9:** Non-sig.	**(ATTSPH-SF):** Sig. Between group effect over time (d=.58)	**DSS:** Significant between group reduction (d=.53)

Symptomatology
CES-D Center for Epidemiologic Studies Depression Scale
ATQ Automatic Thoughts Questionnaire
BDI-II Beck Depression Inventory
SAPS Scale for Assessment of Positive Symptoms
PHQ-9 Patient Health Questionnaire 9
GAD-7 Generalized Anxiety Disorder 7
K10 Kessler Psychological Distress Scale

Mental health literacy
D-Lit Depression Literacy Questionnaire
CBT-Lit CBT Literacy questionnaire
KASI Knowledge About Schizophrenia Interview
A-Lit Anxiety Literacy Questionnaire
DKS Depression Knowledge Survey
RTS Resistance to Treatment Survey

Help seeking scales
AHSQ Actual Help Seeking Questionnaire
GHSQ General Help Seeking Questionnaire
ATTSPH-SF Attitudes Toward Seeking Professional Help-Short form

Stigma
DSS Depression Stigma Scale
GASS Generalized Anxiety Stigma Scale
SOSS Stigma of Suicide Scale
BSDS Bogardus Social Distance Scale

Other
CSI-SF Coping Strategies Inventory Short-Form
RS Resilience Scale
AUDIT Alcohol Use Disorders Identification Test
SWLS Satisfaction With Life Scale
EUROHIS-QOL European Health Interview Survey-Quality of Life
BSDS (MDD & HIV) Bogardus Social Distance scale (Major depression & HIV)
